# Supplementary material for: Spike-phase coupling of subthalamic neurons to posterior perisylvian cortex predicts speech sound accuracy
Source: Nat Commun. 2025 Apr 9;16:3357. doi: 10.1038/s41467-025-58781-8 (PMC11982203; doi:10.1038/s41467-025-58781-8)
Supplement: Supplementary file 1 — Supplementary Information [file 41467_2025_58781_MOESM1_ESM.pdf]

Supplementary Text

Articulation disorders and voice quality – A team of trained speech-language pathologists used a custom MATLAB GUI (github.com/Brain-Modulation-Lab/SpeechCodingApp) to annotate each phone of syllable triplet productions. We adopted three terms that characterized articulatory dimensions (distortion, imprecision, and disfluency) and six terms that characterized voice dimensions (creaky, horse-harsh, voice-break, strain, tremor, and breathy) of speech. A phone was considered *distorted* if it was so indistinct that the SLP could not identify it, *imprecise* if the phoneme was identifiable but lacked clarity and precision. Phones were labeled *disfluent* if any range of disruptions occurred such as blocks, prolongations, and repetitions.

In-silico evaluation of the SPC pipeline – As our work mainly relies on the concept of time occurrence of transient spike-phase coupling (t-SPC) events, we evaluated the ability of our pipeline in identifying genuine SPC events in a reasonable set of parameters constrained by our dataset. We simulated a set of trials (N=80) of sinusoidal oscillations at a specific frequency of interest (f = 8 Hz) for 3 seconds. To avoid phase resetting, we inserted a noise in the phase of the oscillation. We modeled spiking times employing a Poissonian distribution with baseline spiking rate of 25 Hz. To incorporate firing rate modulation, we modulated the baseline spiking rate with a gaussian distribution centered around the peak of the neuron response (Mean = 1 (no modulation), 0.5 (50% reduction), 1.5 (50% increase); SD = 0.2 s). We also defined a ground-truth of SPC in a 0.3 s long window (comparable to our t-SPC events duration) around a preferred phase ($\pi/4$) and defined a modulation of the spiking rate based on the cosine distance of the current phase with respect to the preferred phase. This cosine distance was modulated in such a way that the ratio between the firing rate at the preferred phase and the firing rate at the opposite phase is equal to the effect size (ES)[1], defined as $\left(1+2\sqrt{\mathrm{PPC}}\right)/\left(1-2\sqrt{\mathrm{PPC}}\right)$, which means that a PPC of 0.015 (comparable to what we obtained in our dataset, see Figure S20) would correspond to an ES of 1.65. We then ran the same cluster-based permutation test to extract the significance of SPC and defined the t-SPC. We swept a set of parameters and compared the timing identification of the t-SPC event: true PPC strength (or ES equivalently), the duration of the t-SPC event, the time resolution (number of bins or anchor points), the number of trials which regulates the target window size around each bin, the frequency of coupling, the baseline firing rate, the jitter of the behavioral events (standard deviation across trials), the firing rate modulation, and the sampling rate. In Figure S18A-B we reported an example of SPC computation with a simple moving average window (no adaptation of the window width) and our method without true t-SPC events (ES=1, PPC=0) and a genuine t-SPC event (ES=1.65, PPC=0.015) between 1.7 s and 2 s (red patch). The analysis (Figure S18C) also confirmed that the estimation of the duration of the SPC (black crosses: before cluster-based permutation test, red crosses: after cluster-based permutation test) is reliable for a reasonable set of parameters if a conservative cluster-based permutation is applied. Interestingly, sampling rate is extremely important to get accurate estimations and

only drastic decreasing (~ 0.25) or increasing (> 2.25) modulations of firing rate might bias the timing identification of the t-SPC event.

Detailed explanation of the calculation for equidistant anchor points given a target time resolution – To calculate the number of anchor points between behavior events based on a target resolution, defined as $\frac{B_W}{W}$ (Equation (1)):

$$N_{anchors} = \left\lfloor \frac{\left\lfloor \frac{B_W}{W} * T \right\rfloor + N_{events}}{N_{events} + 1} \right\rfloor \tag{1}$$

where $\lfloor \circ \rfloor$ is the floor function, B_W is the target number of anchor points in a window of duration W (B_W/W is the resolution, e.g., 50 ms), T is the duration of the trial , N_{events}, is the number of relevant behavior events (4 in our case: auditory cue onset, auditory cue offset, speech production onset and speech production offset), $N_{anchors}$ is the number of equidistant anchor points between two events. This calculation ensures continuity between intervals so that the final anchor of each interval matches the first anchor of the subsequent interval. The points are thus structured as: [1-$N_{anchors}$, 2-$N_{anchors}$, 2-$N_{anchors}$, 2-$N_{anchors}$], creating an uninterrupted series of anchor points across the trial.

List of Supplementary Tables

Table S1: Clinical patient information. Summary of the clinical description of the patient cohort, including symptom severity, recordings availability and behavioral performance. M/F: male/female, UPDRS: Unified Parkinson's Disease Rating Scale, NR: value was not recorded in the medical record. We report age and gender in aggregate (mean ± SD) in the Methods section of the manuscript.

ID	Surgery side	UPDRS III OFF	UPDRS III Speech OFF	UPDRS III ON	UPDRS III Speech ON	#task runs	#ECoG strips	MER tracks	#Units	Speech duration, s	# trials	Phonetic accuracy, %
**DBS3001**	Left	25	0	10	0	4	1	MCP	22 (19)	1.29	344	86.61
**DBS3002**	Left	29	1	16	1	2	1	MCP	5 (4)	0.99	360	60.34
**DBS3003**	Left	17	1	3	0	3	2	MCP	13 (13)	1.39	480	77.60
**DBS3004**	Left	61	1	36	2	3	1	MCP	11 (9)	1.35	360	55.00
**DBS3008**	Left	NR	NR	NR	NR	2	1	MCP	11 (10)	1.54	360	74.17
**DBS3010**	Left	34	1	14	1	3	3	MCP	14 (13)	1.16	480	78.96
**DBS3011**	Left	30	1	7	0	3	2	MCP	8 (8)	0.89	480	69.05
**DBS3012**	Left	43	2	26	2	4	2	ACP	13 (11)	1.19	600	84.01
**DBS3014**	Left	30	0	19	0	3	2	MCP	15 (!5)	1.52	480	50.42
**DBS3015**	Left	42	1	24	1	3	1	MCP	9 (9)	1.61	480	87.29
**DBS3017**	Left	34	1	26	1	3	1	MCP	6 (2)	1.59	345	48.83
**DBS3018**	Left	21	1	13	0	3	2	MCP	9 (8)	1.27	480	88.08
**DBS3019**	Left	40	NR	18	NR	3	2	MCP	13 (7)	1.13	360	20.55
**DBS3020**	Left	19	0	15	0	2	2	MCP	7 (7)	1.28	360	26.76
**DBS3022**	Left	38	1	20	1	2	2	MCP	9 (9)	1.05	360	3.89
**DBS3023**	Left	30	2	7	1	3	2	MCP	11 (11)	1.24	360	27.94
**DBS3024**	Left	37	1	27	1	2	2	MCP	11 (8)	1.13	360	69.05
**DBS3026**	Left	50	1	24	1	2	2	MCP	7 (5)	1.23	148	6.40
**DBS3027**	Left	33	0	30	0	2	2	MCP	9 (9)	1.37	360	39.17
**DBS3028**	Left	39	2	29	2	3	2	MCP	18 (16)	1.48	480	88.57
**DBS3029**	Left	NR	NR	NR	NR	2	2	MCP	6 (5)	1.40	240	52.08
**DBS3030**	Left	43	2	30	2	2	2	MCP	5 (5)	2.49	240	56.25
**DBS3031**	Left	39	2	26	2	2	2	MCP	5 (4)	1.26	240	22.38
**DBS3032**	Left	47	1	27	1	3	2	MCP	8 (4)	1.49	360	80.39
**Mean (SD)**	**-**	**35.5 (10.18)**				**2.67 (0.62)**	**1.79 (0.49)**		**24 5 (211)**	**1.35 (0.41)**	**379.88 (99.49)**	**56.41(26.91)**

Table S2: Articulation and voice quality as evaluated by a trained speech-language pathologist. A trained team of speech-language pathologist students inspected the produced audio signals and evaluated the quality of the voice and articulation at the single phoneme level. Please refer to Supplementary Text for a description of each term. Spirantization is considered as a category of imprecise articulation.

	**Articulation disorder terms [%]**			**Voice terms [%]**					
**ID**	**distortion**	**imprecision**	**dysfluency**	**creaky**	**horse-harsh**	**voice-break**	**strain**	**tremor**	**breathy**
**DBS3001**	0.30	11.76	0.05	0	0	0	0	0	2.08
**DBS3002**	0.47	4.30	0	0.09	0	0.09	0	0.28	0.14
**DBS3003**	2.52	3.97	0.09	0.14	0	0.09	0.19	0.42	0.61
**DBS3004**	2.41	17.31	0.74	0	0	0	1.25	0.88	1.16
**DBS3008**	1.57	3.70	0.28	21.39	0	0	0	0.74	0
**DBS3010**	0.07	8.58	0	0.03	0	0.10	0	0.21	6.53
**DBS3011**	0.08	3.02	0	4.33	0	0.04	0	0.04	0.08
**DBS3012**	1.80	10.40	0	2.14	0	0.15	0.11	0.23	0.26
**DBS3014**	0.07	7.47	0.56	0.31	0	0	0	0	0
**DBS3015**	0.38	4.97	0	0.42	0	0	0	65.76	0.80
**DBS3017**	4.54	6.10	0	1.02	0	0.47	1.41	0.47	3.83
**DBS3018**	0.48	5.52	0	0.33	0	0.04	0	0.66	0.11
**DBS3019**	1.12	6.46	0.33	0	0	0.26	1.12	0.13	13.18
**DBS3020**	0.42	5.02	0.05	5.12	0	0.05	0	1.88	0.56
**DBS3022**	0.69	12.36	0	0	0	0	0.05	0.09	0.19
**DBS3023**	0.41	3.19	0.33	4.66	0	1.06	0	0.74	0
**DBS3024**	0.53	3.20	0.10	17.38	0	0.10	0.10	0.38	0.48
**DBS3026**	5.60	24.53	0.27	0	0	0	0	3.33	0.53
**DBS3027**	0.97	3.98	0.32	12.36	0	0.05	0	2.73	0.42
**DBS3028**	0.24	3.21	0.24	0.71	0	0.08	0	5.75	0.75
**DBS3029**	0.63	3.54	0.07	3.75	0	0	0.49	0	0.28
**DBS3030**	0.63	5.14	0.35	0.76	0	0	0.56	5.35	7.71
**DBS3031**	1.75	5.63	0.08	0.48	0	0	0	0.32	2.30
**DBS3032**	0.48	0.91	0	2.25	0	0.48	0	0	4.77
**Mean (SD)**	1.17 (1.40)	6.84 (5.27)	0.16 (0.20)	3.24 (5.73)	0 (0)	0.13 (0.14)	0.22 (0.43)	3.77 (13.30)	1.95 (3.20)

Table S3: Number of pairs, participants, STN neurons and ECoG contacts included in the main analysis for each ROI.

ROI	PreCG	MFG	SMG	STG	PostCG	SCG	pars O.
**# pairs**	3547	3177	2716	2557	2440	1807	1178
**# participants**	22	17	18	22	20	19	9
**# STN neurons**	22	22	22	28	28	28	22
**# ECoG contacts**	110	91	85	51	75	67	53

Table S4: Frequency-wise spatial centroid and peak of spatial density of SPC topographies. Results are reported for the cortex and subthalamic nucleus.

**Cortex**							
	**Centroid**				**Peak**		
	**X [mm]**	**Y [mm]**	**Z [mm]**		**X [mm]**	**Y [mm]**	**Z [mm]**
θ	-67.51	-27.91	24.86		-69.73	-49.94	12.89
α	-62.23	-13.04	30.4		-68.86	-38.46	24.12
β	-65.27	-10.14	28.41		-71.14	-13.78	29.80
γ_L	-62.66	-5.45	26.74		-52.67	10.63	39.76
γ_H	-62.05	-3.92	26.94		-49.35	16.02	41.50
**STN**							
	**Centroid**				**Peak**		
	**X [mm]**	**Y [mm]**	**Z [mm]**		**X [mm]**	**Y [mm]**	**Z [mm]**
θ	-13.54	-15.38	-6.3		-13.65	-15.23	-5.4
α	-13.23	-15.26	-8.07		-13.09	-15.63	-8.59
β	-13.32	-14.16	-7.64		-13.8	-13.08	-8.51
γ_L	-13.12	-15.11	-7.70		-15.85	-16.2	-6.61
γ_H	-12.81	-15.02	-7.88		-12.33	-14.27	-11.77

Table S5: Number of pairs, participants, STN neurons and ECoG contacts included in the error analysis (refer to Methods for details) for each ROI.

ROI	PreCG	MFG	SMG	STG	PostCG	SCG	pars O.
**# pairs**	126	84	169	117	201	66	0
**# participants**	13	8	11	9	13	7	0
**# STN neurons**	15	14	14	12	13	8	0
**# ECoG contacts**	38	41	47	41	50	31	0

List of Supplementary Figures

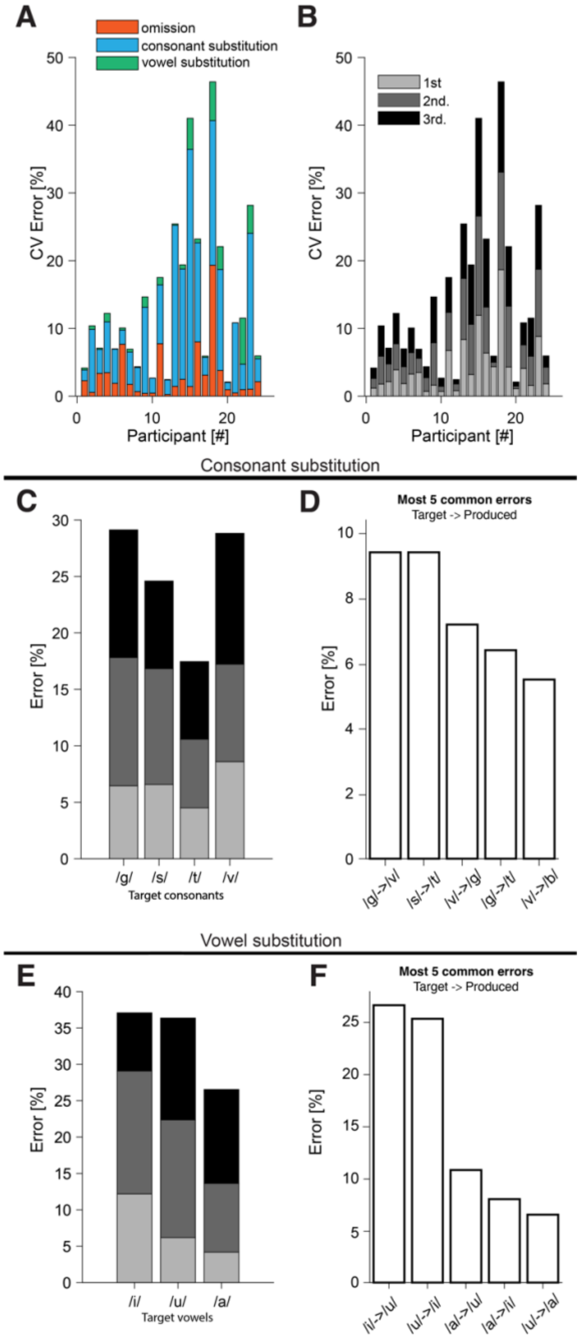

Figure S1: Characterization of speech errors. **(A)** Percentage of speech error types across participants (#errors / # phonemes). Errors were categorized in phoneme omission (orange), consonant substitution (light blue) and vowel substitution (green). **(B)** Proportion of speech errors in the first (light gray), second (gray) and third (black) consonant-vowel (CV) syllable. **(C)** Proportion of consonant substitution error for each target consonant. **(D)** List of 5 common consonant substitution errors across participants. **(E)** Proportion of vowel substitution error for each target vowel. **(F)** List of 5 common vowel substitution errors across participants. Source data are provided as a Source Data file.

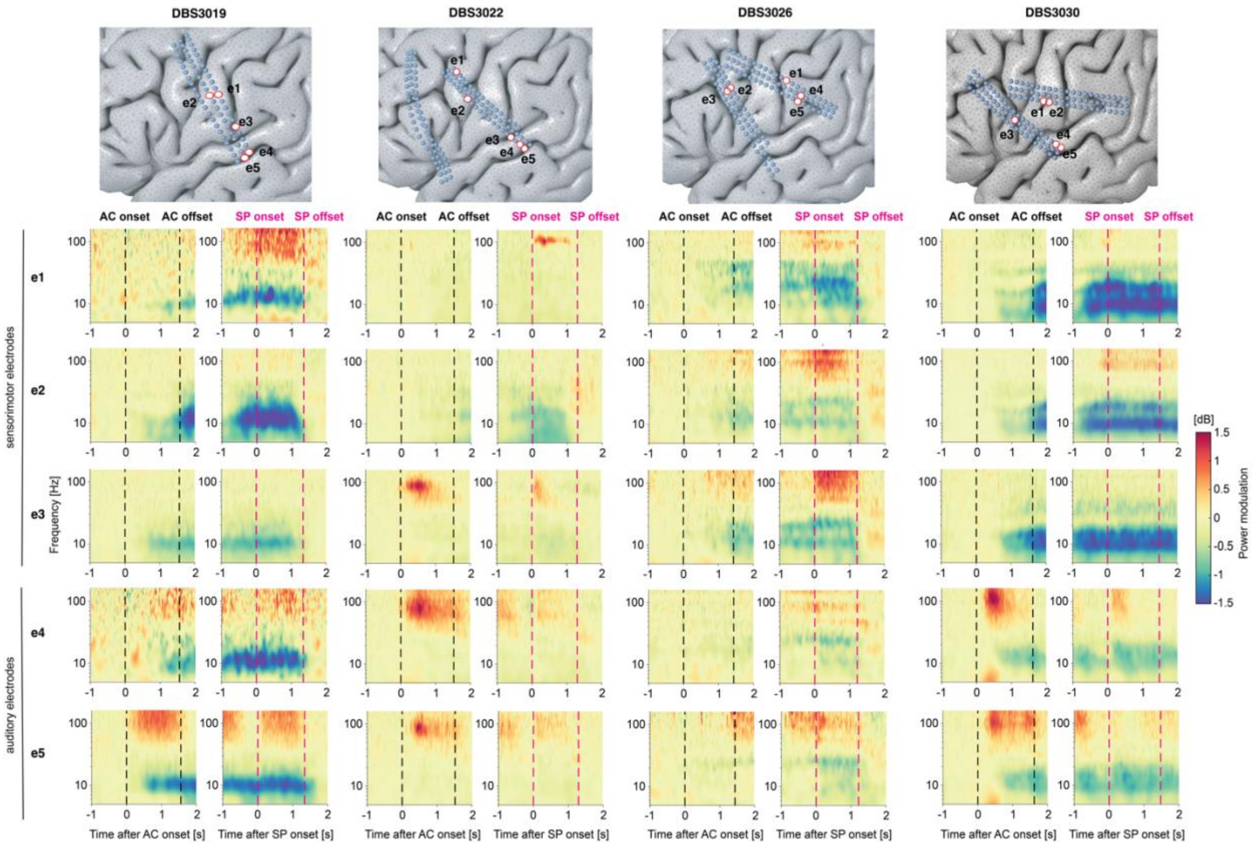

Figure S2: Electrocorticographic channels from four exemplary participants. Electrode locations and time-frequency spectrograms locked to auditory cue (AC) (black dashed line) onset and speech production (SP) (magenta dashed line) onset are depicted for each participant. Two representative sensorimotor electrodes (e1, e2) and three auditory electrodes (e3, e4, e5) are shown. Time-frequency spectrograms are normalized with respect to the baseline (during the inter-trial interval (ITI)) and expressed as dB.

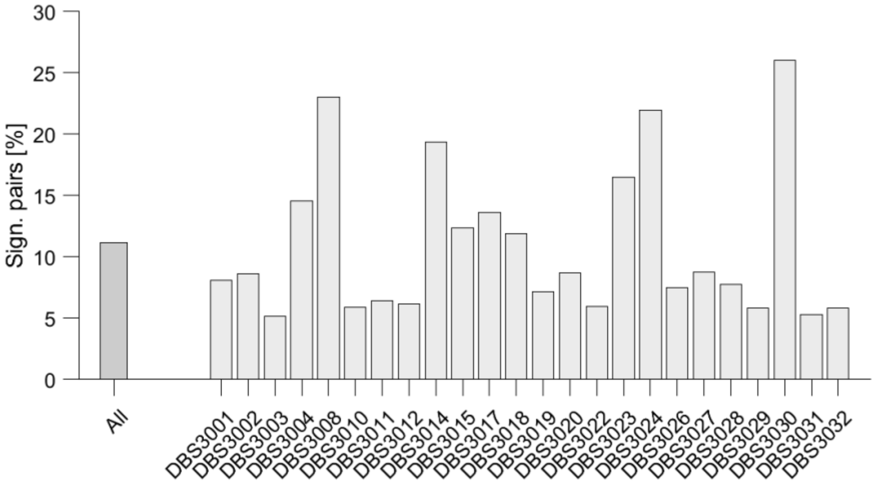

Figure S3: Percentage of significant SPC pairs across participants. Results for each participant (light gray) and pooled across all participants (dark gray) are shown.

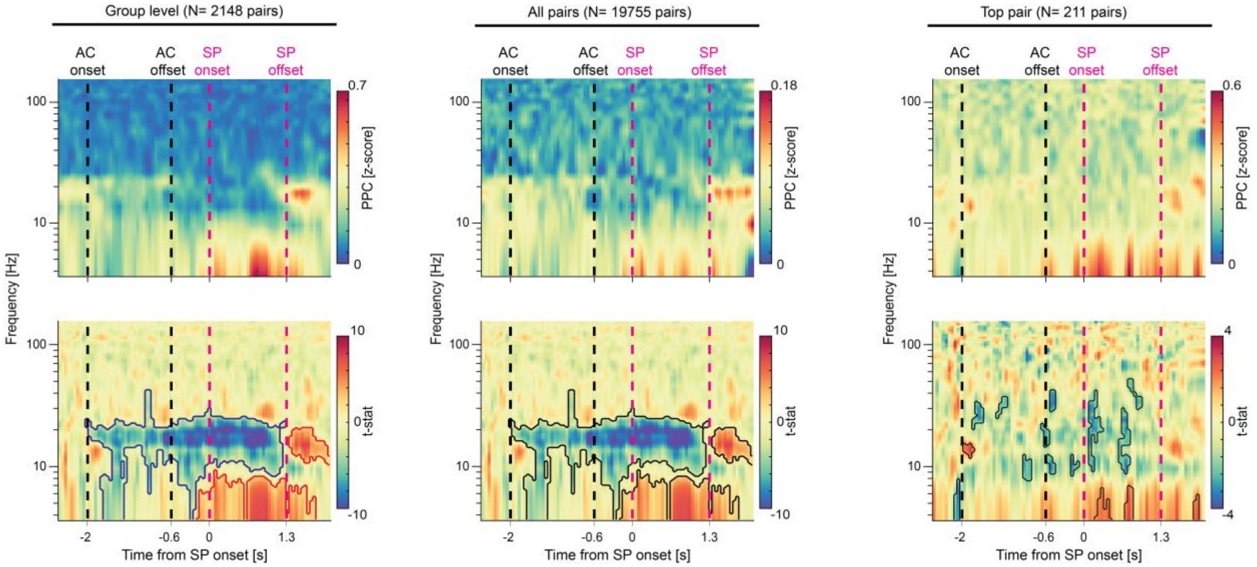

Figure S4: Comparison of average SPC maps across different subsets of pairs. (Top) Results are consistent whether we averaged only significant SPC maps (N = 2148, same panel of Figure 2A), all pairs (N = 19755) or the most significant SPC map for each unit. (Bottom) Same as Top with the group-level statistical test (t-stat) of the significance of the z-score PPC with respect to the baseline. Red and blue lines contour regions of significant SPC increase or decrease, respectively. Auditory cue (AC) (black dashed line) onset and speech production (magenta dashed line) windows are represented. Source data are provided as a Source Data file.

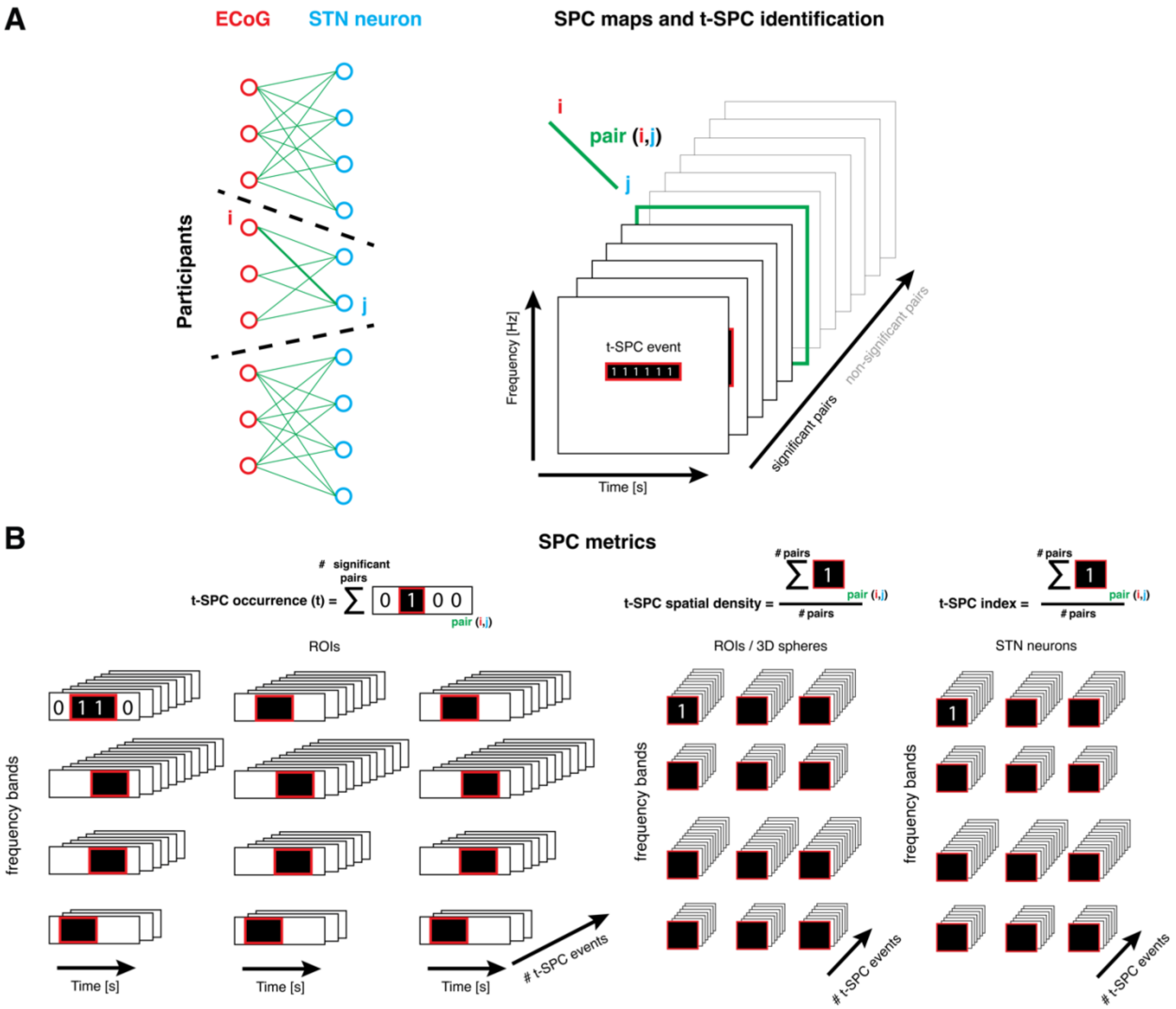

Figure S5: Overview of the SPC metrics. (**A**) For each participant we calculated the spike-phase coupling in each possible pair (all-to-all schema, green lines) of ECoG contacts (red circle) and STN neurons (cyan circle) and obtained a time-frequency SPC map. A cluster-based permutation test was used to identify significant transient spike-phase coupling (t-SPC) events (black boxes with red contour) and binarize each SPC map. A pair was deemed to be significant if it contained one or more t-SPC events. (**B**) We grouped and combined these binarized SPC maps in different ways to extract different metrics: t-SPC occurrence was used to evaluate the time occurrence of t-SPC events and their degree of aggregation over time, t-SPC spatial density was used to evaluate the spatial distribution over cortex and STN (grouping by ROI or 3D sphere, refer to Methods for details), and t-SPC index was used for analyses in which the unit of observation was the STN neuron (grouping by neuron). All metrics are expressed as percentage.

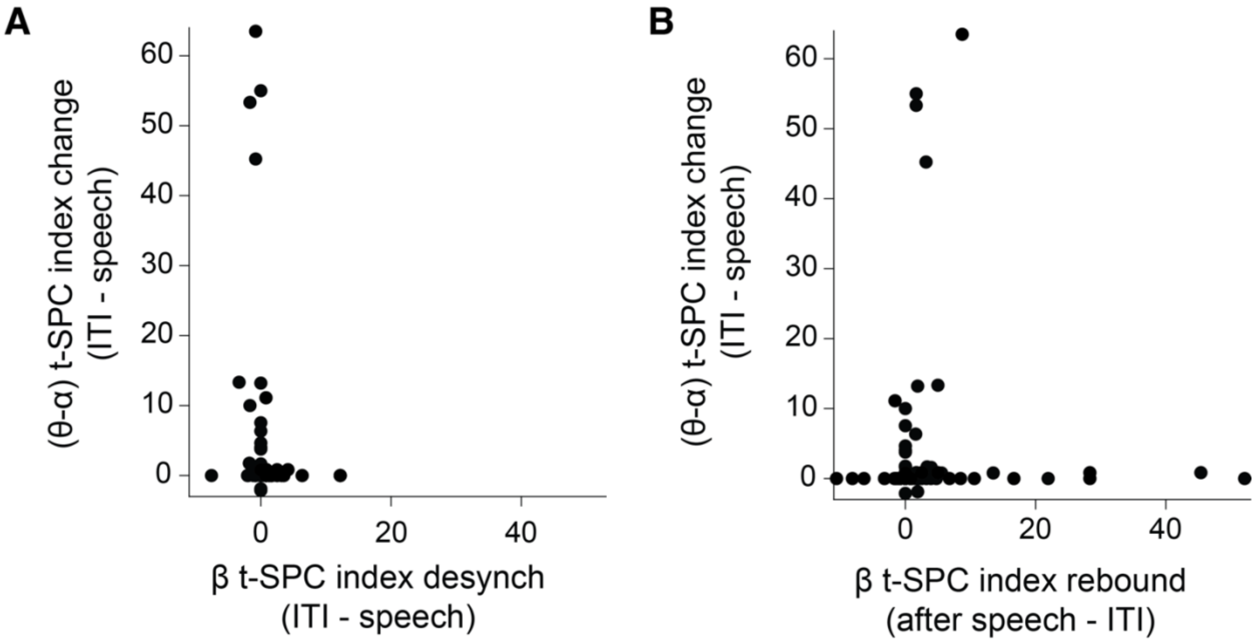

Figure S6: Neurons are highly specific to only one frequency band.
Each dot represents the t-SPC index (ratio of the number of t-SPC events and pairs for each STN neuron) increase in the (θ-α) range plotted against to the decrease in the β-range during the speech production window (**A**), and the increase (rebound) in the β-range after the speech termination (**B**). The observation unit is the neuron (211 dots). The orthogonal relationship highlights the independence between t-SPC density changes in different frequency bands.

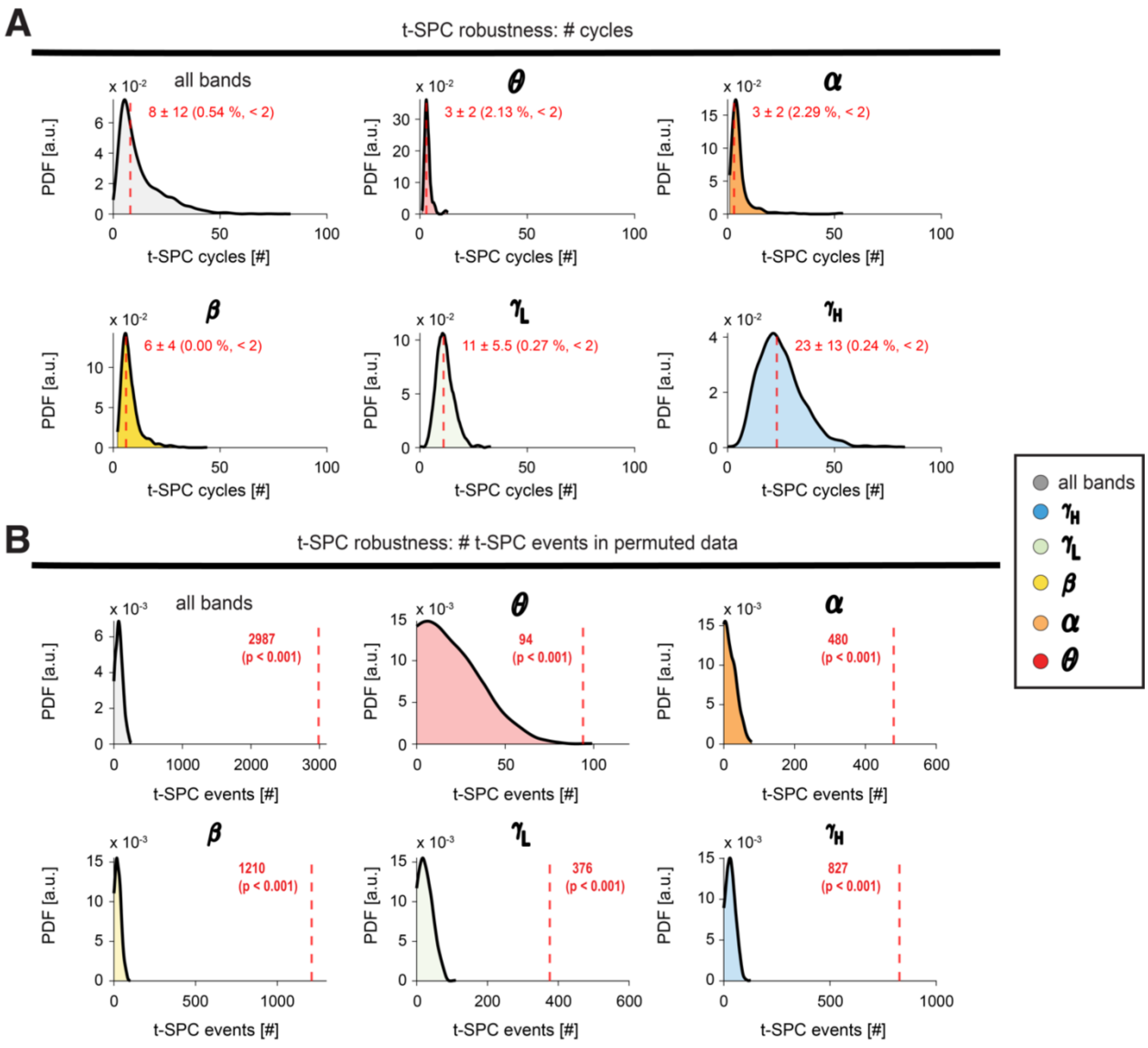

Figure S7: Robustness analysis of the t-SPC event identification. **(A)** Distribution of the number of cycles that span the duration of the t-SPC events in all bands and grouped by frequency band. We also report the percentage of t-SPC events spanning less than 2 cycles (see Methods). Results are reported as median ± interquartile range **(B)** Distribution of the number of t-SPC events observed in the permuted SPC maps. Red dashed line depicts the median of the experimentally observed number of t-SPC events for each band. We applied the t-max correction across frequency bands in each panel to control for multiple comparisons. θ (red), α (dark orange), β (yellow), γ_L (green) and γ_H (blue). Source data are provided as a Source Data file.

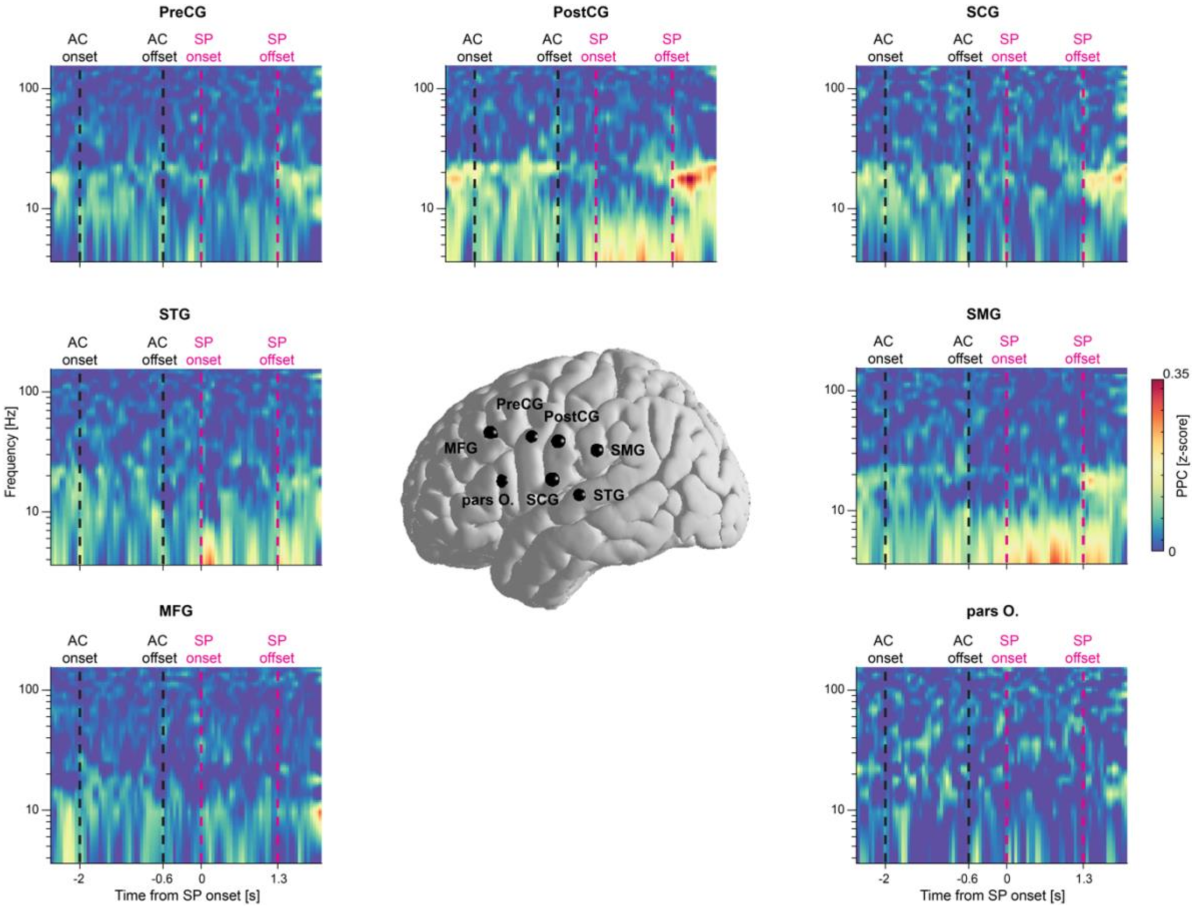

Figure S8: Average of the spike-phase coupling (SPC) maps for all spike-phase coupling pairs (N = 2148) in each region of interest (ROI). The pairwise-phase consistency (PPC) index is compared to the permutation distribution and expressed as z-score. Black and magenta vertical dashed lines denote auditory cue (AC) and speech production (SP) windows. The centroid location (black spheres) of the ECoG contacts included in this analysis is shown for each ROI. Refer to Table S3 for the number of pairs included in each ROI.

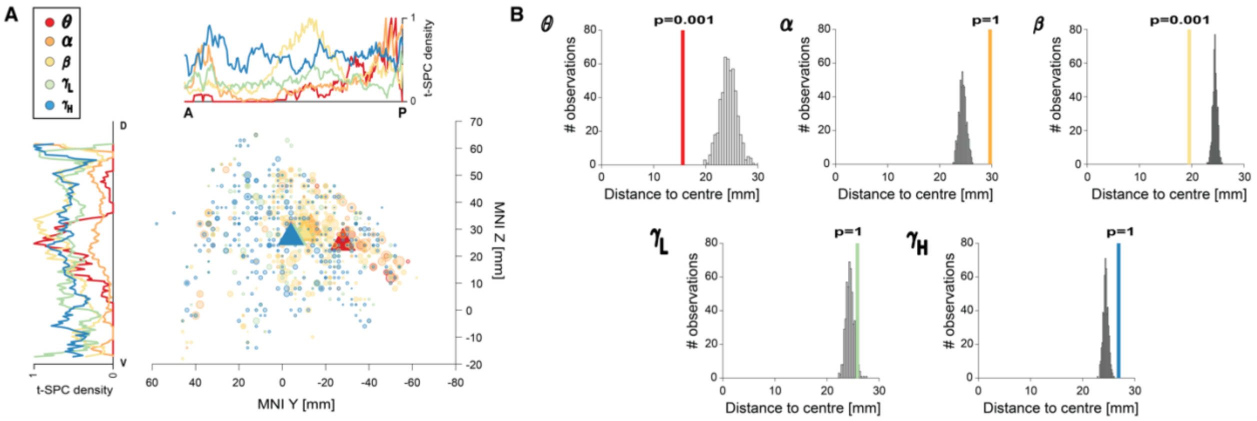

Figure S9: Spatial aggregation of spectral SPC cortical topographies. (**A**) Localization of the spike-phase coupling topography of all frequency bands in the sagittal plane (MNI Z vs Y coordinates). Inset plots show the average spatial density mapped along the dorso (D) – ventral (V) and anterio (A) – posterior (P) axes. (**B**) Results of the spatial aggregation analysis to test the focality of the distribution of the SPC topographies. Histogram of the permutation distances and the observed distance (vertical line) are illustrated. Triangles depict the spatial centroid of each topography. θ (red), α (dark orange), β (yellow), γ_L (green) and γ_H (blue). We applied the t-max correction across frequency bands in (**B**) to control for multiple comparisons (1000 permutations). Source data are provided as a Source Data file.

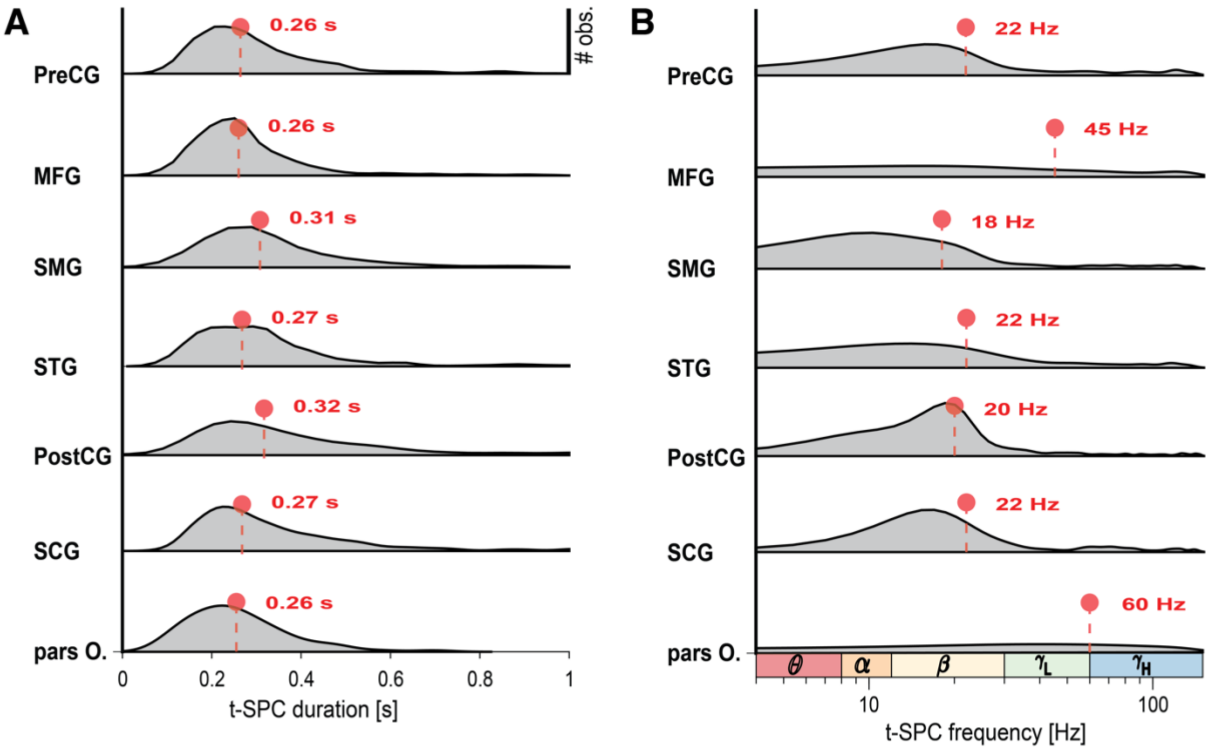

Figure S10: Cortical heterogeneity of t-SPC events duration and frequency. Distribution of the t-SPC duration (**A**) and t-SPC frequency (**B**) in seven cortical regions of interest. To augment the readability of the t-SPC frequency distribution, we adopted the logarithmic scale. Dot and dashed lines depict the median of the distribution. θ (red), α (dark orange), β (yellow), γ_L (green) and γ_H (blue). List of cortical regions of interests: Precentral gyrus (PreCG), Postcentral gyrus (PostCG), Supramarginal gyrus (SMG), Superior temporal gyrus (STG), Middle frontal gyrus (MFG), and the orbital part of the inferior frontal gyrus (pars O.). We applied the t-max correction across ROIs in each panel to control for multiple comparisons. Source data are provided as a Source Data file.

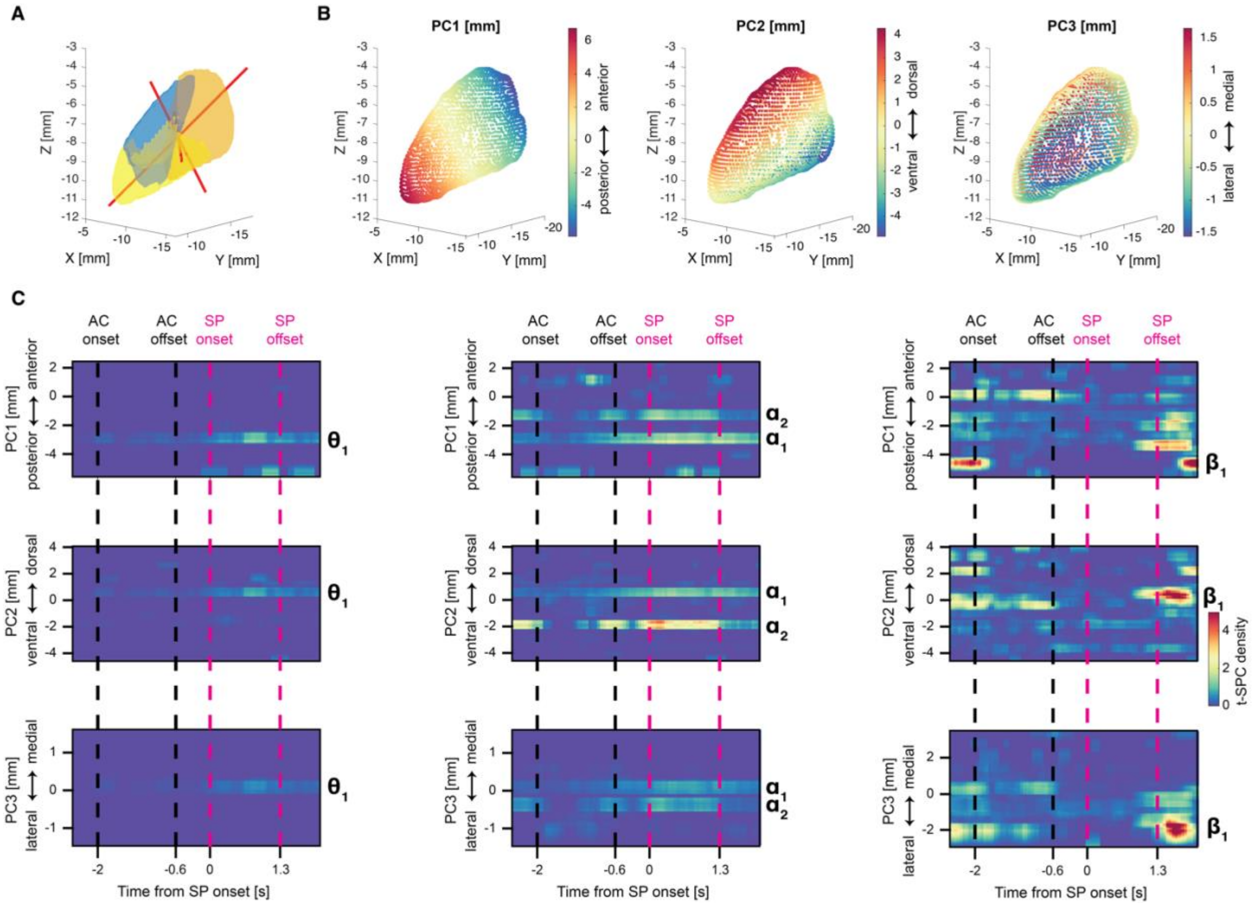

Figure S11: A new reference of frame to represent STN coordinates. **(A)** Illustration of the Subthalamic nucleus and its territorial subdivision (motor: orange, associative: yellow and limbic: blue), as depicted by the DISTAL atlas[2]. Direction of the principal component axes is shown as red line. Length of the line is proportional to the explained variance. (**B**) Each principal component has a specific anatomical interpretation: (PC1: anterior-posterior axis, PC2: dorso-ventral axis and PC3: medio-lateral). Note that principal component scores represent actual physical distances in mm. (**C**) Spatial density of the t-SPC events mapped along the three principal component axes. The anatomical reference of frame shows the relative orientation between the dorsal (D), lateral (L) and posterior (P) directions and the first three principal components directions (PC1: anterior-posterior axis, PC2: dorso-ventral axis and PC3: medio-lateral). Black and magenta vertical dashed lines denote auditory cue (AC) and speech production (SP) windows. Cross indicates the spatial centroid of the t-SPC event locations. θ_1, α_1, α_2 and β_1 depict the location of peaks of the t-SPC spatial density. Note that principal component scores represent actual physical distances in mm. Source data are provided as a Source Data file.

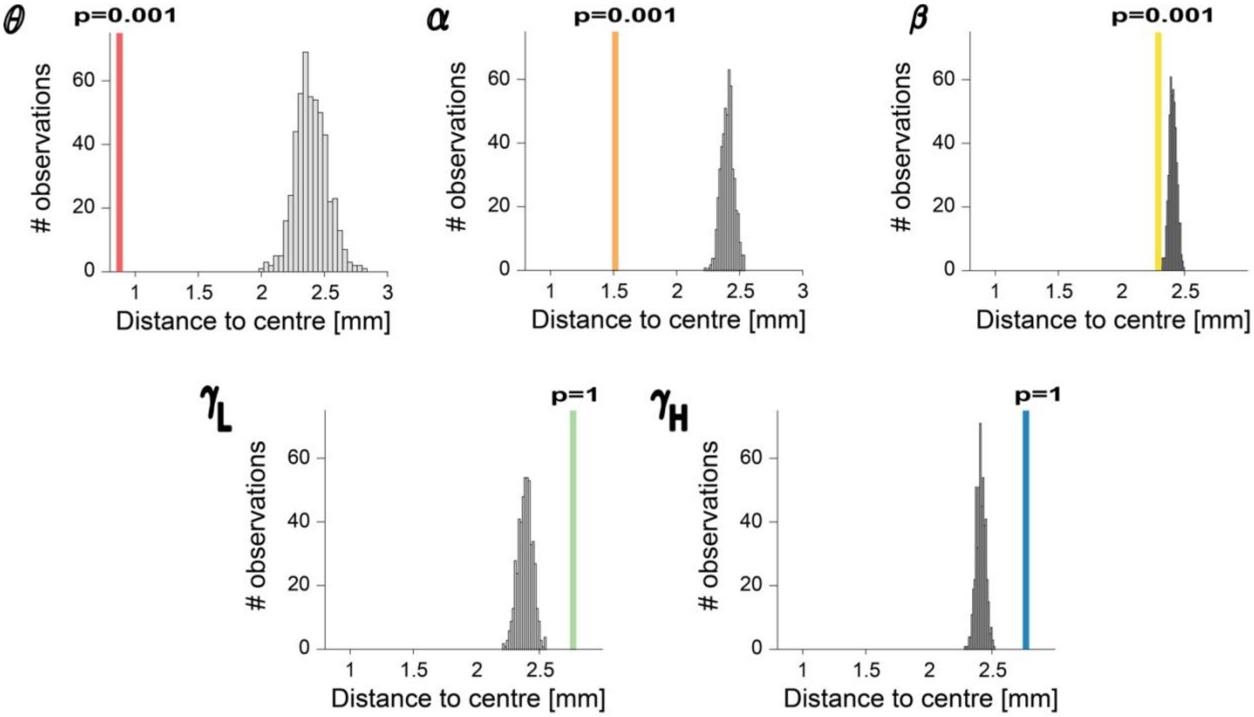

Figure S12: Spatial aggregation of spectral SPC STN topographies. Results of the spatial aggregation analysis to test the spatial dispersion of SPC within the STN. Histogram of the permutation distances and the observed distance (vertical line) are illustrated. Triangles depict the spatial centroid of each topography. θ (red), α (dark orange), β (yellow), γ_L (green) and γ_H (blue). We applied the t-max correction across frequency bands to control for multiple comparisons. Source data are provided as a Source Data file.

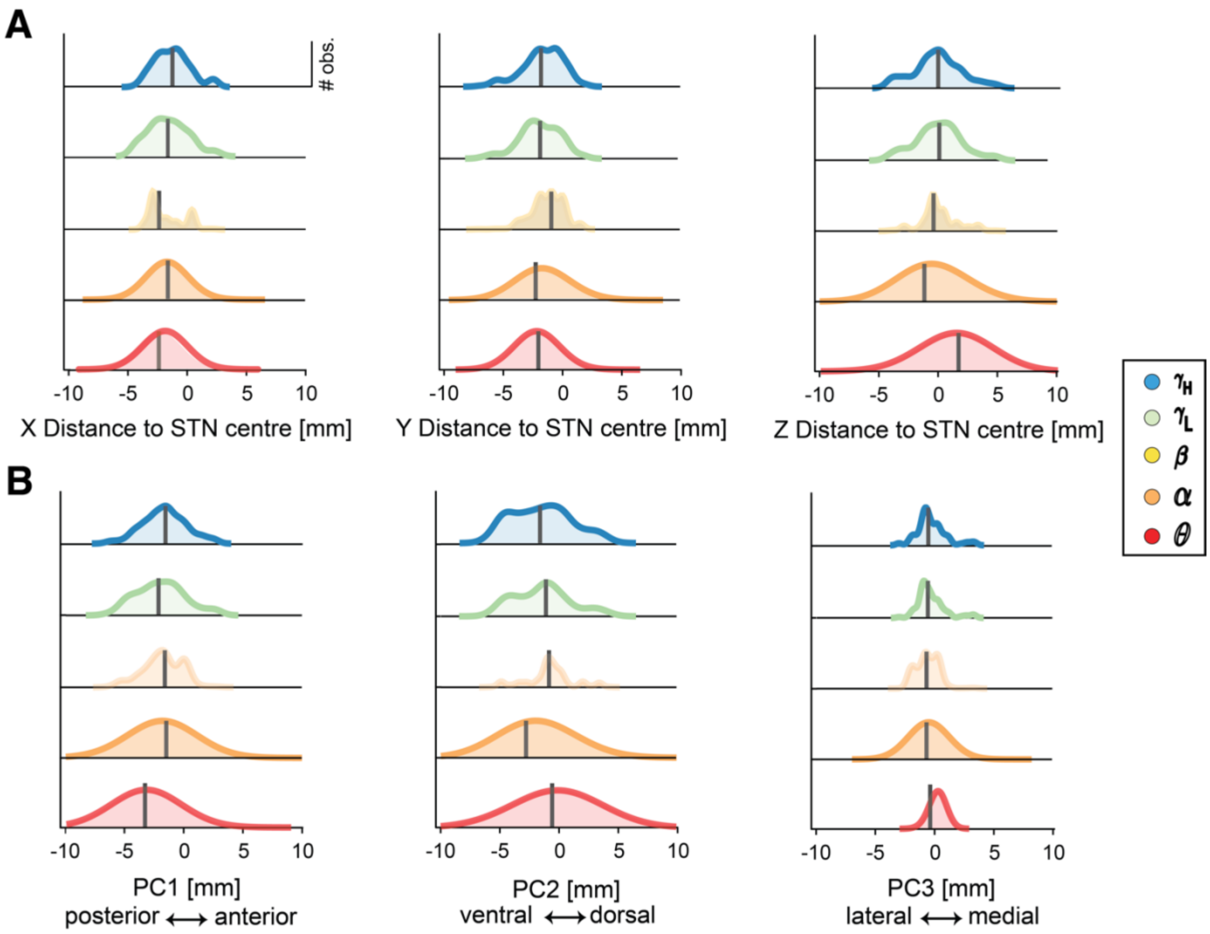

Figure S13: Localization of the t-SPC events in the STN. Distribution of the location of the t-SPC events in MNI coordinates (**A**) and principal component axes (**B**) across all frequency bands. Note that principal component scores represent actual physical distances in mm. Black line depicts the median of the distribution. θ (red), α (dark orange), β (yellow), γ_L (green) and γ_H (blue). We applied the t-max correction across frequency bands in each panel to control for multiple comparisons. Source data are provided as a Source Data file.

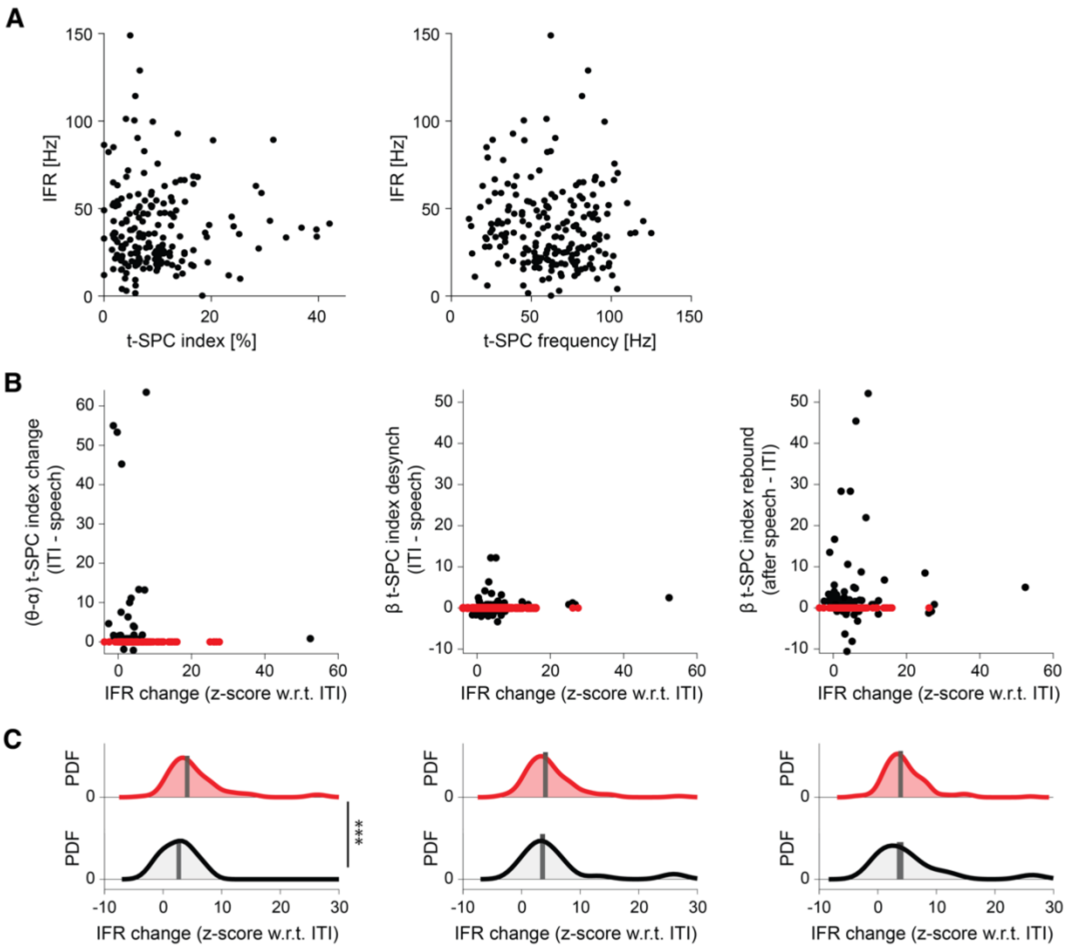

Figure S14: Relationship between IFR modulation and SPC changes during speech production. (**A**) Relationship between the baseline instantaneous firing rate and (Left) the t-SPC index (i.e., ratio between # t-SPC events and # pairs) and (Right) the t-SPC frequency centroid across neurons. (**B**) Each dot represents the t-SPC index increase (speech - ITI) in the (θ-α) range and decrease (ITI - speech) in the β-range during the speech production window, and the increase (rebound; after speech - ITI) in the β-range after the speech termination plotted against to the z-score (peak change) of the instantaneous firing rate with respect to the ITI. The observation unit is the neuron (211 dots). Red dots highlight neurons with no change in the t-SPC spatial density. (**C**) Distribution of the z-score change of the instantaneous firing rate in neurons with (black) and without (red) changes in the t-SPC spatial density. Asterisks indicate the significance (***, $p_{perm} < 0.001$) of the permutation test that assesses significant difference between the two distributions. Black line depicts the median of the distribution. Source data are provided as a Source Data file.

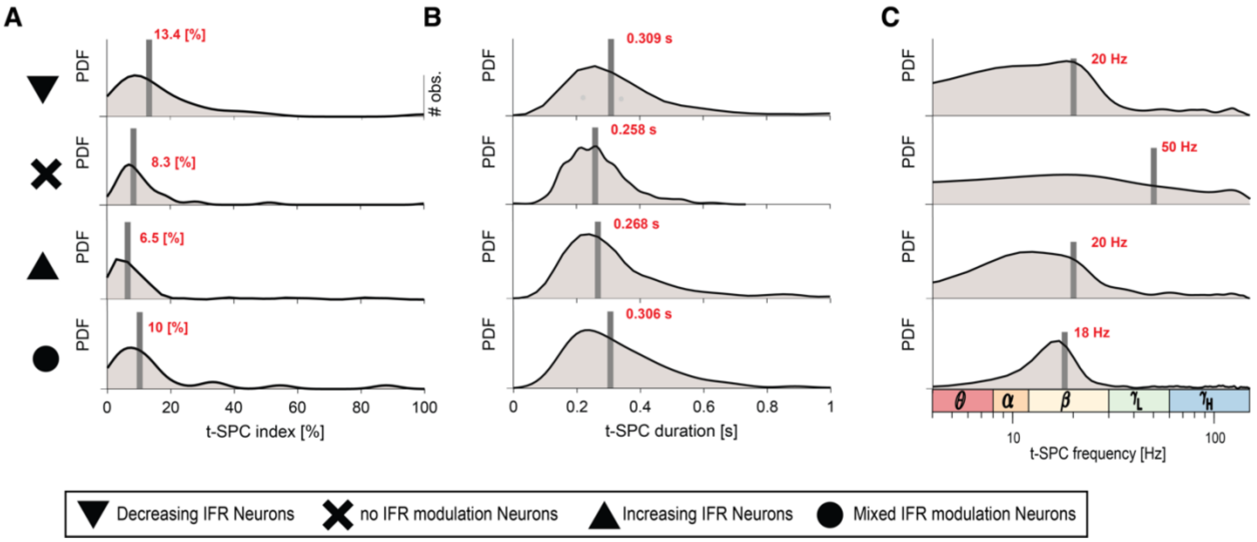

Figure S15: t-SPC event characteristics across four categories of neurons based on the IFR modulation during the syllable triplet repetition task. Distribution of the t-SPC index (**A**), t-SPC duration (**B**) and t-SPC frequency (**C**) in each IFR category. Note in (**C**) the low-pass profile of the t-SPC frequency distribution in decreasing IFR neurons, band-pass profile in increasing IFR neurons and even more localized in mixed IFR modulation neurons. To augment the readability of the t-SPC frequency distribution, we adopted the logarithmic scale. List of IFR categories: Decreasing IFR neurons (downward triangle), no IFR modulation neurons (cross), Increasing IFR neurons (upward triangle) and Mixed IFR modulation neurons (circle). Black line depicts the median of the distribution. Source data are provided as a Source Data file.

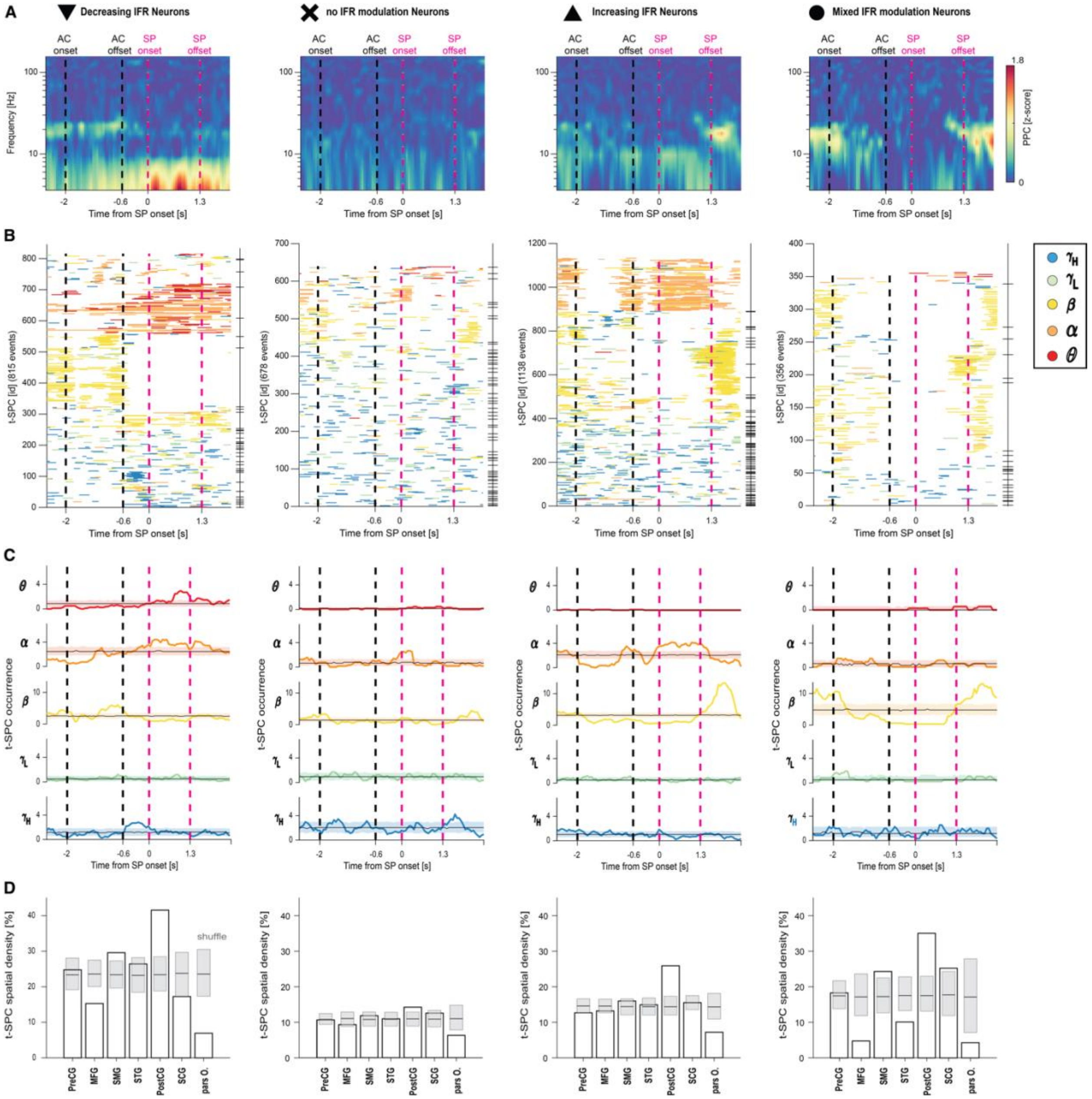

Figure S16: Spike-phase coupling changes in different instantaneous firing rate categories of neurons. (**A**) Average of the spike-phase coupling (SPC) maps for significant spike-phase coupling pairs (N = 2148). The pairwise-phase consistency (PPC) index is compared to the permutation distribution and expressed as z-score. (**B**) List of the t-SPC events (N = 2987) sorted by increasing frequency centroid. Horizontal ticks indicate different neurons. (**C**) t-SPC event occurrence grouped by frequency band. Shaded areas illustrate the 5th and 95th percentiles of the permutation distribution for the aggregation test. (**D**) Overall spatial density of the t-SPC events in seven regions of interest, as derived from the Destrieux atlas[3]. Dark gray boxes indicate the 5th and 95th percentile of the permutation distribution for the spatial preference test. Regions with spatial density higher or lower than the permutation distribution are labelled as high or low spatial preference. Black and magenta vertical dashed lines denote auditory cue (AC) and speech production (SP) windows. θ (red), α (dark orange), β (yellow), γ_L (green) and γ_H (blue). We applied the t-max correction across ROIs in (**D**) to control for multiple comparisons. Source data are provided as a Source Data file.

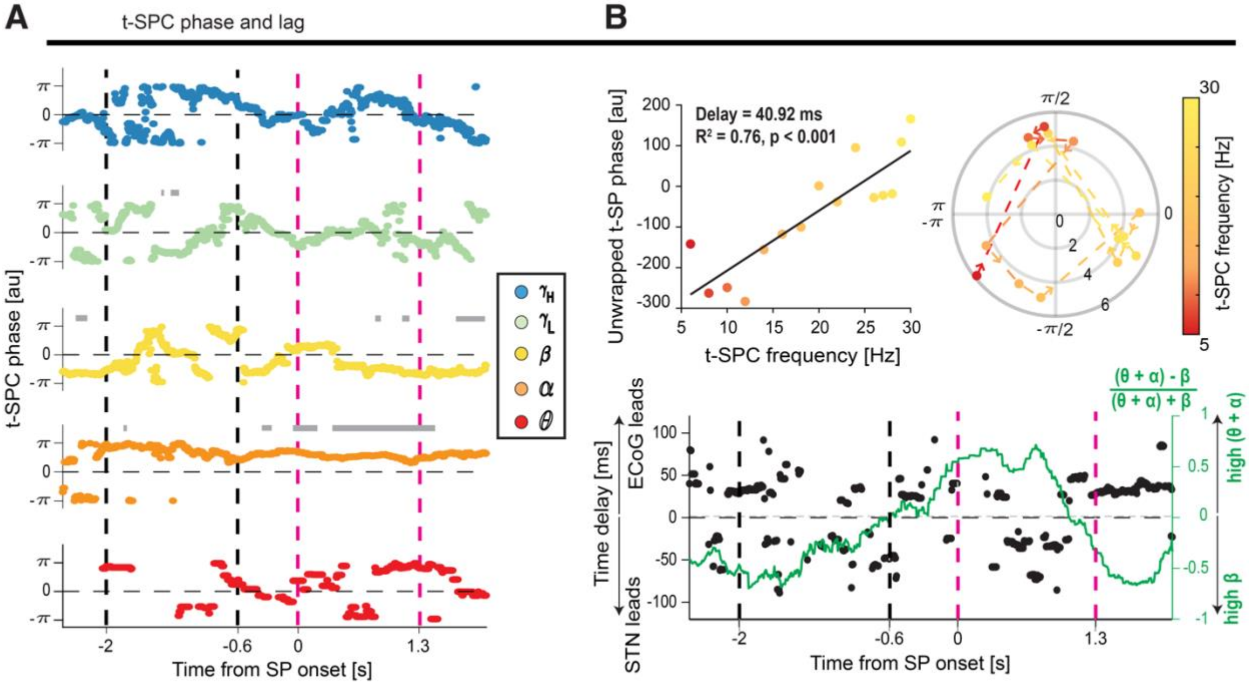

Figure S17: Phase and time delay relationship **(A)** Average preferred phase of firing during the t-SPC events across frequency bands. Gray bar depicts time bins with significant non-uniformity of the preferred phase across all pairs. θ (red), α (dark orange), β (yellow), γ_L (green) and γ_H (blue). (**B**) Unwrapped phase values show their progression on a linear scale. The time delay derived from the gradient of the linear fit was 40.92 ms. A permutation test confirmed the significance of the relationship between unwrapped phases and frequency. The sign of the time delays defines the leading activity: positive (ECoG leads STN) and negative (STN leads ECoG). Circular plot shows mean angle (position on circle) and strength of SPC (PPC) of pairs that are significantly locked at frequencies from 5 to 30 Hz (as indicated by the color scale). Time-resolved estimation of the time delay superimposed with the relative time (green line, see **Time delay analysis** in Methods for details) occurrence of (θ-α) and β t-SPC events, expressed as a contrast between -1 and 1. Black and magenta vertical dashed lines denote auditory cue (AC) and speech production (SP) windows Source data are provided as a Source Data file.

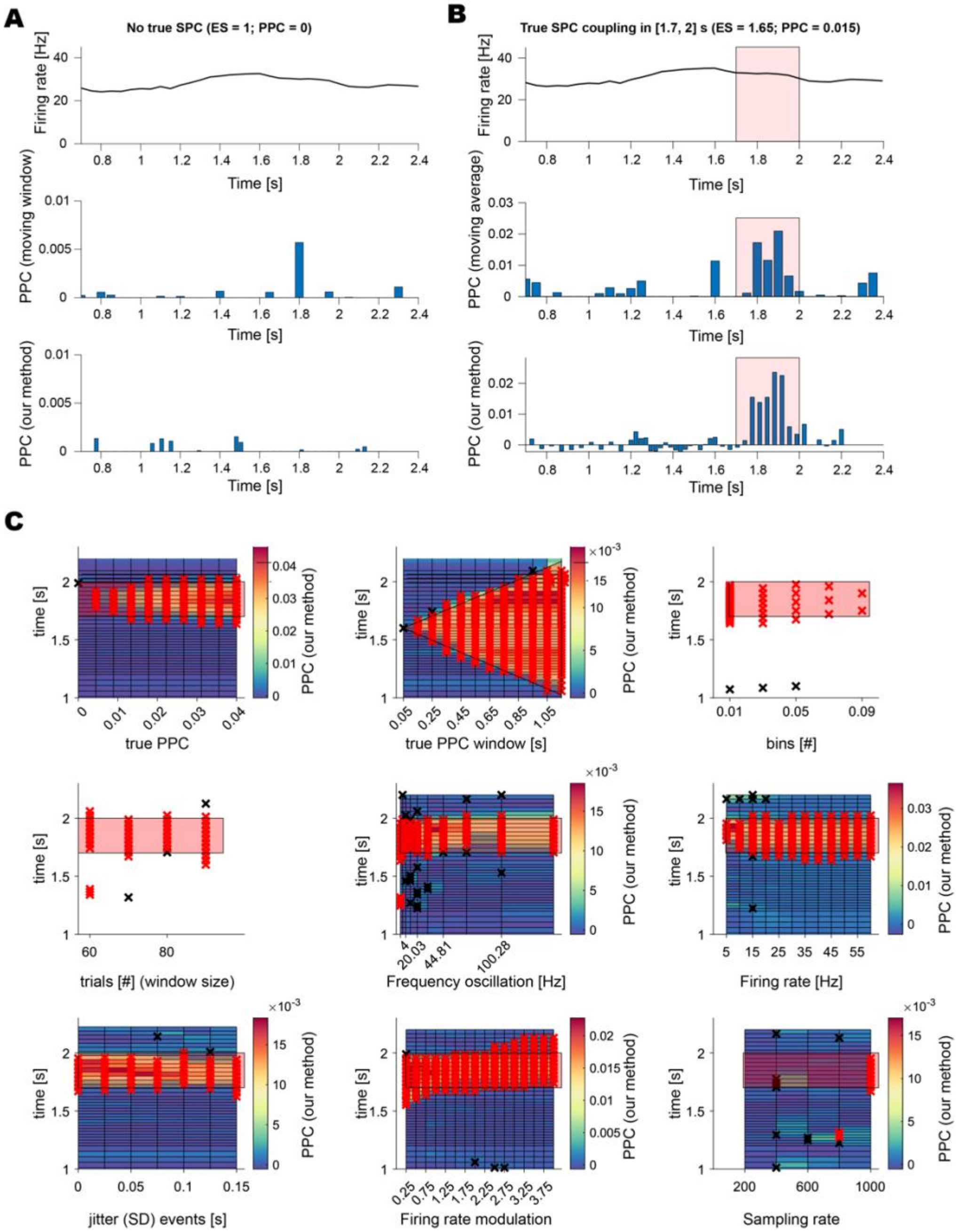

Figure S18: Our SPC pipeline can accurately estimate transient events of spike-phase coupling. (**A**) (Top) Example of simulation of a neuron with increasing firing rate and no spike-phase coupling (ES = 1, (pairwise phase-consistency) PPC = 0). (Center) A moving window (fixed 150 ms width and 25 ms of time resolution) was used to compute the PPC and, hence, the SPC strength. (Bottom) Our SPC pipeline was used to estimate SPC strength. (**B**) Same as (**A**) with a true spike-phase coupling event (ES = 1.65, PPC = 0.015) in a 0.3 s long window (red patch). (**C**) Our method can accurately detect (before (black cross) and after (red cross) cluster-based permutation test) a short period of true spike-phase coupling (red patch) for a reasonable set of parameters if a conservative cluster-based permutation is applied. Set of parameters: true PPC strength (or ES equivalently), the duration of the t-SPC event, the time resolution (number of bins), the number of trials which regulates the target window size around each bin, the frequency of coupling, the baseline firing rate, the jitter of the behavioral events, the firing rate modulation, and the sampling rate. Sampling rate is extremely important to get accurate estimations and only drastic decreasing (~ 0.25) or increasing (> 2.25) modulations of firing rate might bias the timing identification of the t-SPC event.

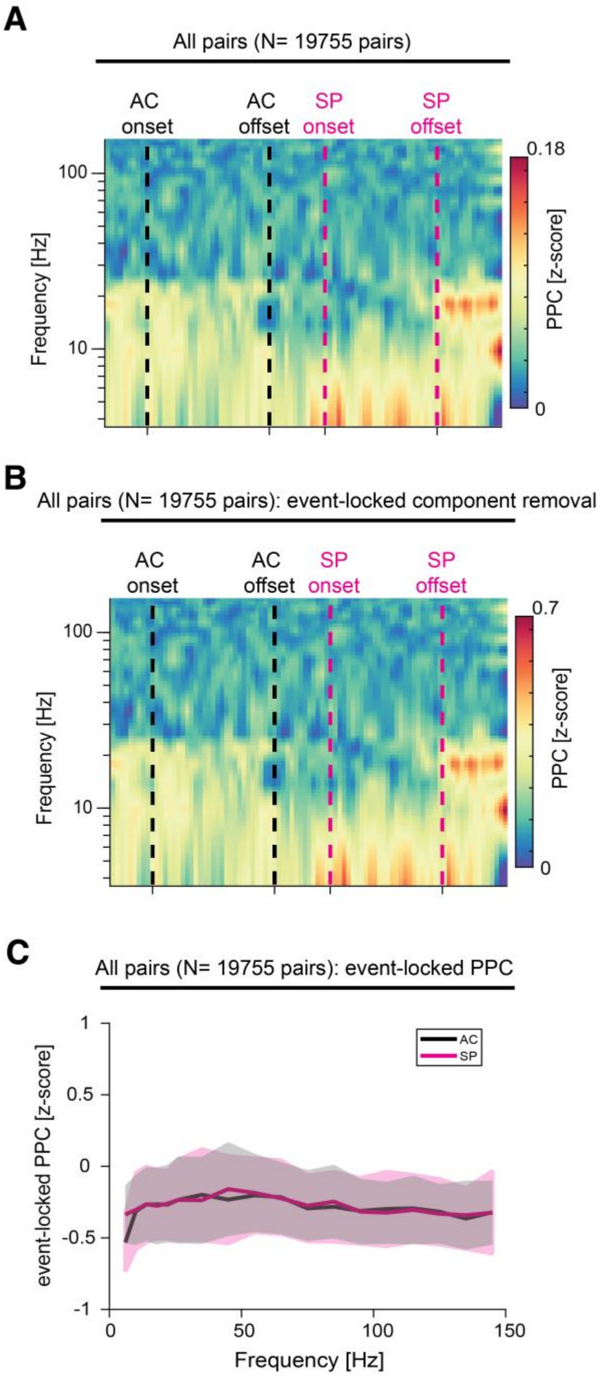

Figure S19: Event-locked component does not influence the spike-phase coupling estimation. Results are consistent whether we averaged all SPC maps (N = 19755, same panel of Figure S3B) before (**A**) and after (**B**) the removal of the event-locked component. Auditory cue (AC) (black dashed line) and speech production (magenta dashed line) windows are represented. (**C**) Quantification of phase-reset using the same method as used for assessing SPC. We computed event locked SPC by considering as spikes the onset of the auditory cue (black) and speech production (red). Shaded area shows the 95% confidence interval across all pairs (N = 19755).

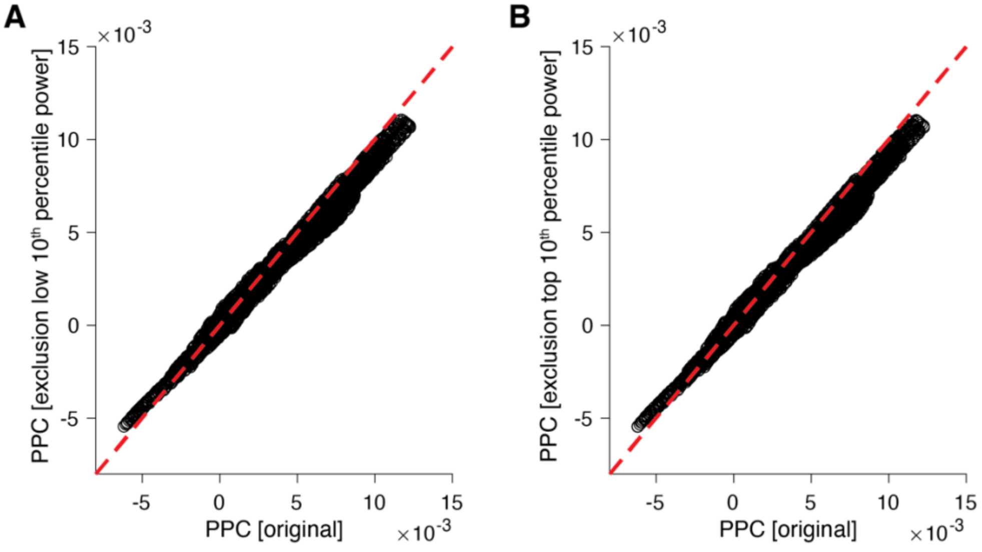

Figure S20: Calculation of the raw PPC metric is not affected by extremely high or lower power episodes. We repeated the calculation of the PPC metric (Methods, SPC pipeline) before and after the removal of periods with low amplitude oscillations (low 10th percentile) (**A**) and high amplitude (high 10th percentile) (**B**). The red dashed line depicts the identity line.

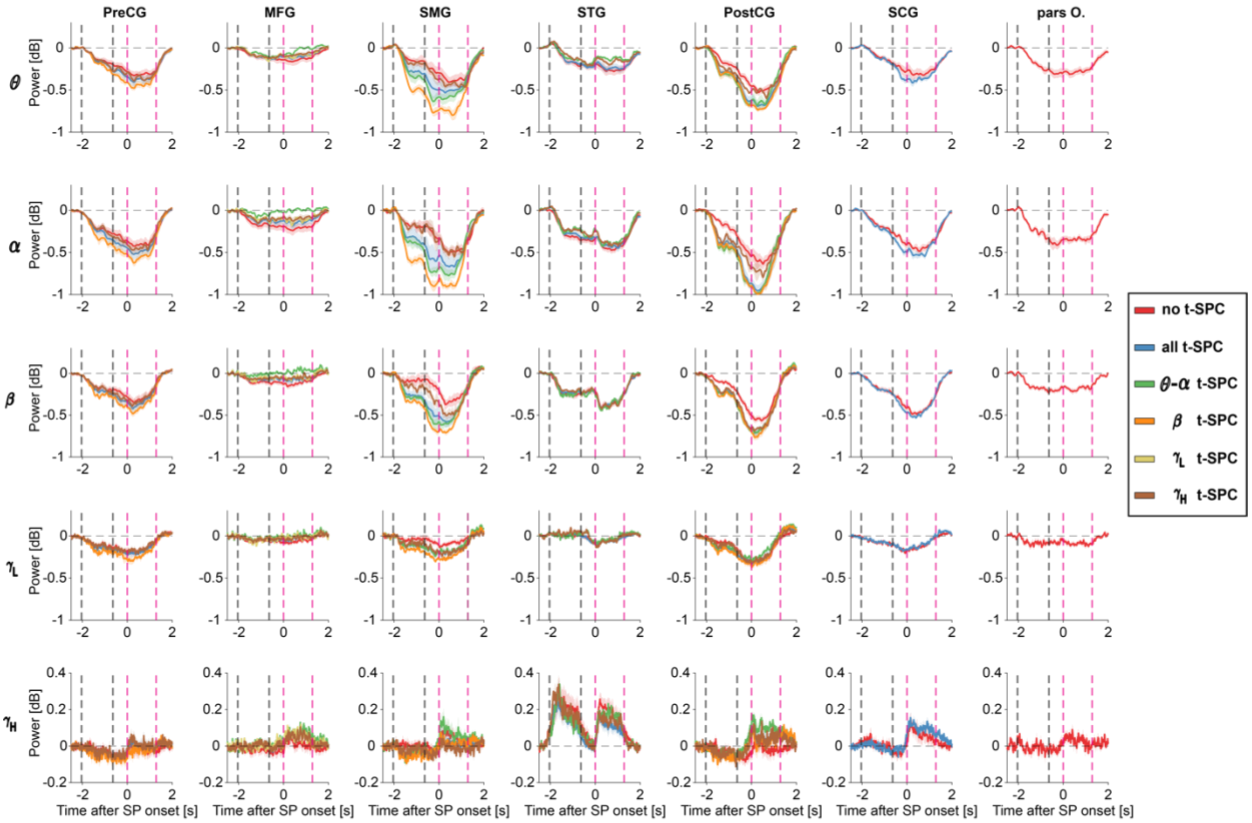

Figure S21: Cortical ECoG contacts with significant SPC in the PostCG, PreCG and SMG are more task responsive. Comparison of the speech-related power modulation in ECoG contacts without significant t-SPC events (red) and those with significant t-SPC events across any frequency band (blue), as well as within specific bands: θ-α (green), β (orange), γ_L (yellow), and γ_H (magenta). Data are grouped by region of interest (columns) and frequency bands (rows). Only conditions with at least 20 ECoG channels are shown. Black and magenta vertical dashed lines denote auditory cue and speech production (SP) windows. Shaded areas illustrate the 5th and 95th percentile of the bootstrapped distribution of the mean.

Supplementary References

1. Vinck, M., van Wingerden, M., Womelsdorf, T., Fries, P., and Pennartz, C.M.A. (2010). The pairwise phase consistency: a bias-free measure of rhythmic neuronal synchronization. Neuroimage *51*, 112–122. https://doi.org/10.1016/j.neuroimage.2010.01.073.

2. Ewert, S., Plettig, P., Li, N., Chakravarty, M.M., Collins, D.L., Herrington, T.M., Kühn, A.A., and Horn, A. (2018). Toward defining deep brain stimulation targets in MNI space: A subcortical atlas based on multimodal MRI, histology and structural connectivity. NeuroImage *170*, 271–282. https://doi.org/10.1016/j.neuroimage.2017.05.015.

3. Destrieux, C., Fischl, B., Dale, A., and Halgren, E. (2010). Automatic parcellation of human cortical gyri and sulci using standard anatomical nomenclature. NeuroImage *53*, 1–15. https://doi.org/10.1016/j.neuroimage.2010.06.010.
