# Supplementary material for: Spike-phase coupling of subthalamic neurons to posterior perisylvian cortex predicts speech sound accuracy
Source: Nat Commun. 2025 Apr 9;16:3357. doi: 10.1038/s41467-025-58781-8 (PMC11982203; doi:10.1038/s41467-025-58781-8)
Supplement: Supplementary file 2 — Reporting Summary [file 41467_2025_58781_MOESM2_ESM.pdf]

nature portfolio

Corresponding author(s): Matteo Vissani, R. Mark Richardson

Last updated by author(s): 02/25/2025

Reporting Summary

Nature Portfolio wishes to improve the reproducibility of the work that we publish. This form provides structure for consistency and transparency in reporting. For further information on Nature Portfolio policies, see our Editorial Policies and the Editorial Policy Checklist.

Statistics

For all statistical analyses, confirm that the following items are present in the figure legend, table legend, main text, or Methods section.

n/a	Confirmed	
☐	☒	The exact sample size (*n*) for each experimental group/condition, given as a discrete number and unit of measurement
☐	☒	A statement on whether measurements were taken from distinct samples or whether the same sample was measured repeatedly
☐	☒	The statistical test(s) used AND whether they are one- or two-sided<br>*Only common tests should be described solely by name; describe more complex techniques in the Methods section.*
☐	☒	A description of all covariates tested
☐	☒	A description of any assumptions or corrections, such as tests of normality and adjustment for multiple comparisons
☐	☒	A full description of the statistical parameters including central tendency (e.g. means) or other basic estimates (e.g. regression coefficient) AND variation (e.g. standard deviation) or associated estimates of uncertainty (e.g. confidence intervals)
☐	☒	For null hypothesis testing, the test statistic (e.g. *F*, *t*, *r*) with confidence intervals, effect sizes, degrees of freedom and *P* value noted<br>*Give P values as exact values whenever suitable.*
☒	☐	For Bayesian analysis, information on the choice of priors and Markov chain Monte Carlo settings
☒	☐	For hierarchical and complex designs, identification of the appropriate level for tests and full reporting of outcomes
☒	☐	Estimates of effect sizes (e.g. Cohen's *d*, Pearson's *r*), indicating how they were calculated

Our web collection on statistics for biologists contains articles on many of the points above.

Software and code

Policy information about availability of computer code

Data collection	Electrocorticographic (ECoG) signals were acquired with high-density (54 or 63 contacts) strips (PMT Cortact), referenced to a sterile stainless-steel subdermal needle electrode placed on the scalp and acquired at 30kHz with a Grapevine Neural Interface Processor equipped with Micro2 Front Ends (Ripple LLC, Salt Lake City, UT, USA). Microelectrode recordings (MERs) were acquired with the Neuro-Omega recording system (Alpha-Omega Engineering, Nof HaGalil, Israel) using parylene insulated tungsten microelectrodes (25 µm in diameter, 100 µm in length). See detailed description in Method section. The original dataset is available for download upon request at https://dabi.loni.usc.edu/dsi/1U01NS098969.
Data analysis	ECoG preprocessing was performed using custom code based on the Fieldtrip toolbox implemented in Matlab 2022b, available at (github.com/Brain-Modulation-Lab/bml). Spike sorting was performed using Plexon (https://plexon.com/products/offline-sorter/). Audio signals were processed using Praat (https://www.fon.hum.uva.nl/praat/) and a custom Matlab interface (github.com/Brain-Modulation-Lab/SpeechCodingApp). The localization of ECoG contacts was determined using the Randazzo localization method (github.com/Brain-Modulation-Lab/ECoG_localization) and anatomical labels were assigned using the Destrieux atlas (https://surfer.nmr.mgh.harvard.edu/fswiki/CorticalParcellation). MERs contacts were localized using costum Matlab scripts (github.com/Brain-Modulation-Lab/Lead_MER) built open LeadDBS v3.0 (https://www.lead-dbs.org). Anatomical plots were created using SurfIce (https://www.nitrc.org/projects/surfice). The RainCloud library was used to visualize and plot data distributions (https://github.com/RainCloudPlots/RainCloudPlots). Statistics for circular data was run using the CircStat Toolbox (https://github.com/circstat/circstat-matlab). Code is available for download at Zenodo (https://doi.org/10.5281/zenodo.12610957) and Github (https://github.com/Brain-Modulation-Lab/code_SPC_ECoG_STN_Speech).

For manuscripts utilizing custom algorithms or software that are central to the research but not yet described in published literature, software must be made available to editors and reviewers. We strongly encourage code deposition in a community repository (e.g. GitHub). See the Nature Portfolio guidelines for submitting code & software for further information.

Data

Policy information about availability of data

All manuscripts must include a data availability statement. This statement should provide the following information, where applicable:

- Accession codes, unique identifiers, or web links for publicly available datasets
- A description of any restrictions on data availability
- For clinical datasets or third party data, please ensure that the statement adheres to our policy

The data of this study is hosted in the Data Archive BRAIN Initiative (DABI, https://dabi.loni.usc.edu/dsi/1U01NS098969) and is available upon request. No participant identifiable information will be disclosed. The datasets generated and/or analyzed and the statistical tests used during the current study are attached as Source Data and Supplementary Data files.

Research involving human participants, their data, or biological material

Policy information about studies with human participants or human data. See also policy information about sex, gender (identity/presentation), and sexual orientation and race, ethnicity and racism.

Reporting on sex and gender	The methods section contains the self-identified gender in aggregate. No statistical analyses with regard to gender or sex speciific groups have been performed as this was not relevant to the research question in focus in this manuscript.
Reporting on race, ethnicity, or other socially relevant groupings	We neither used nor reported any information about race, ethnicity, or other socially relevant grouping. We did not expect to find relevant differences with the regards of the research question in focus in this manuscript.
Population characteristics	Our population consists of 24 participants (20 males and 4 females, age: 65.4 ± 7.1 years) with Parkinson's Disease undergoing awake stereotactic neurosurgery for implantation of deep brain stimulation electrodes in the subthalamic nucleus . Clinical details are available in the Supplementary Table 1.
Recruitment	Participants undergoing invasive microelectrode recordings for clinical reasons during awake deep brain stimulation surgery were recruited and consented to participate in the research study. ECoG strips were additionally temporally placed through the standard burr hole as part of an IRB approved research protocol. All participants who were capable of and willing to participate in the task, spoke English and had a sufficient level of cognition were offered participation. Dopaminergic medication was withdrawn the night before surgery.
Ethics oversight	All procedures were approved by the University of Pittsburgh Institutional Review Board (IRB Protocol #PRO13110420) and all participants provided informed consent to participate in the study. All participants signed an informed consent prior to study participation. Participants received no compensation in exchange for their participation in the trials and studies.

Note that full information on the approval of the study protocol must also be provided in the manuscript.

Field-specific reporting

Please select the one below that is the best fit for your research. If you are not sure, read the appropriate sections before making your selection.

- [x] Life sciences
- [ ] Behavioural & social sciences
- [ ] Ecological, evolutionary & environmental sciences

For a reference copy of the document with all sections, see nature.com/documents/nr-reporting-summary-flat.pdf

Life sciences study design

All studies must disclose on these points even when the disclosure is negative.

Sample size	Our sample size consists of 211 neurons recorded from 24 patients. No statistical power analysis was used to determine sample size a priori.
Data exclusions	Trials with conspicuous high-power artifacts were identified using an automatic data cleaning procedure. Trials with time segments flagged as artifactual were discarded and channels with more than 30% of artifactual time bins were not included in the analysis. Neurons with at least 10 trials with stable firing rate were included in the main analysis. In the speech sound error analysis we only included neurons with at least 10 trials per condition. The method section contains detailed descriptions of the exclusion criteria.
Replication	The analysis were performed at the single pair (ECoG channel - neuron), single ECoG channel and single neuron level. The effects reported were replicated across 24 patients.
Randomization	The speech production task was designed to collect speech utterances and no experimental conditions were introduced. The speech sound accuracy was used to split our dataset as a within-subject design in which all participants contained accurate and error trials. We performed permutation testing were appropriate to ensure statistical validity of our results. For circular data we preferred to use the CircStat toolbox.
Blinding	Participants were aware we were trying to study speech in basal ganglia but they did not know our specific hypothesis. No experimental conditions were included in the speech production task design. Blinding was no relevant for the collection and analysis of the data. All dataset was collected and analyzed offline.

Reporting for specific materials, systems and methods

We require information from authors about some types of materials, experimental systems and methods used in many studies. Here, indicate whether each material, system or method listed is relevant to your study. If you are not sure if a list item applies to your research, read the appropriate section before selecting a response.

Materials & experimental systems

n/a	Involved in the study
☒	☐ Antibodies
☒	☐ Eukaryotic cell lines
☒	☐ Palaeontology and archaeology
☒	☐ Animals and other organisms
☒	☐ Clinical data
☒	☐ Dual use research of concern
☒	☐ Plants

Methods

n/a	Involved in the study
☒	☐ ChIP-seq
☒	☐ Flow cytometry
☒	☐ MRI-based neuroimaging

Plants

Seed stocks	*Report on the source of all seed stocks or other plant material used. If applicable, state the seed stock centre and catalogue number. If plant specimens were collected from the field, describe the collection location, date and sampling procedures.*
Novel plant genotypes	*Describe the methods by which all novel plant genotypes were produced. This includes those generated by transgenic approaches, gene editing, chemical/radiation-based mutagenesis and hybridization. For transgenic lines, describe the transformation method, the number of independent lines analyzed and the generation upon which experiments were performed. For gene-edited lines, describe the editor used, the endogenous sequence targeted for editing, the targeting guide RNA sequence (if applicable) and how the editor was applied.*
Authentication	*Describe any authentication procedures for each seed stock used or novel genotype generated. Describe any experiments used to assess the effect of a mutation and, where applicable, how potential secondary effects (e.g. second site T-DNA insertions, mosiacism, off-target gene editing) were examined.*
